# Global insights into high temperature and drought stress regulated genes by RNA-Seq in economically important oilseed crop *Brassica juncea*

**DOI:** 10.1186/s12870-014-0405-1

**Published:** 2015-01-21

**Authors:** Ankur R Bhardwaj, Gopal Joshi, Bharti Kukreja, Vidhi Malik, Priyanka Arora, Ritu Pandey, Rohit N Shukla, Kiran G Bankar, Surekha Katiyar-Agarwal, Shailendra Goel, Arun Jagannath, Amar Kumar, Manu Agarwal

**Affiliations:** Department of Botany, University of Delhi Main Campus, Delhi, 110007 India; Department of Plant Molecular Biology, University of Delhi South Campus, Delhi, 110021 India; Bionivid Technology [P] Ltd, Bangalore, 560043 India

**Keywords:** *Brassica juncea*, Transcriptome, High temperature stress, Drought stress, Differential gene expression, Transcription factors, Kinases, Gene ontologies and pathways

## Abstract

**Background:**

*Brassica juncea* var. Varuna is an economically important oilseed crop of family Brassicaceae which is vulnerable to abiotic stresses at specific stages in its life cycle. Till date no attempts have been made to elucidate genome-wide changes in its transcriptome against high temperature or drought stress. To gain global insights into genes, transcription factors and kinases regulated by these stresses and to explore information on coding transcripts that are associated with traits of agronomic importance, we utilized a combinatorial approach of next generation sequencing and *de-novo* assembly to discover *B. juncea* transcriptome associated with high temperature and drought stresses.

**Results:**

We constructed and sequenced three transcriptome libraries namely *Brassica* control (BC), *Brassica* high temperature stress (BHS) and *Brassica* drought stress (BDS). More than 180 million purity filtered reads were generated which were processed through quality parameters and high quality reads were assembled *de-novo* using SOAPdenovo assembler. A total of 77750 unique transcripts were identified out of which 69,245 (89%) were annotated with high confidence. We established a subset of 19110 transcripts, which were differentially regulated by either high temperature and/or drought stress. Furthermore, 886 and 2834 transcripts that code for transcription factors and kinases, respectively, were also identified. Many of these were responsive to high temperature, drought or both stresses. Maximum number of up-regulated transcription factors in high temperature and drought stress belonged to heat shock factors (HSFs) and dehydration responsive element-binding (DREB) families, respectively. We also identified 239 metabolic pathways, which were perturbed during high temperature and drought treatments. Analysis of gene ontologies associated with differentially regulated genes forecasted their involvement in diverse biological processes.

**Conclusions:**

Our study provides first comprehensive discovery of *B. juncea* transcriptome under high temperature and drought stress conditions. Transcriptome resource generated in this study will enhance our understanding on the molecular mechanisms involved in defining the response of *B. juncea* against two important abiotic stresses. Furthermore this information would benefit designing of efficient crop improvement strategies for tolerance against conditions of high temperature regimes and water scarcity.

**Electronic supplementary material:**

The online version of this article (doi:10.1186/s12870-014-0405-1) contains supplementary material, which is available to authorized users.

## Background

The cellular activities are in a continuous state of dynamism and one of the most notable activities in a cell that exemplifies it is gene transcription. Genetic message embedded in the transcripts is translated into proteins that execute predetermined cellular processes. Additionally, some of the transcripts are not translated, but still have the ability to regulate the transcriptional and post transcriptional processes [1-3]. The immediate response of a cell on imposition of a detrimental stress is to take evasive action, which is exhibited by a substantial shutdown of transcription. Concurrently, transcripts of genes, that can mitigate stress injury starts accumulating, the products of which either provide instant protection or salvage the stress-damaged components. Therefore, a large number of studies have focused on the identification of transcripts that are regulated by stress, as they provide a framework for biotechnological approaches to alleviate stress injuries and thereby can be used to make stress tolerant organisms [3-6]. Present understanding of plant response to abiotic stresses reveals that withstanding an adverse condition is a multigenic trait and breeding approaches based on the available germplasm variability has led to significant success in developing environmentally hardy plants [4,5]. In addition to the breeding approaches, overexpression of candidate genes and upstream transcriptional regulators has been widely used to introduce tolerance against abiotic stresses [6]. Because of the multigenic nature of the trait, it is important to collate information on all the molecular factors that orchestrate together to constitute a cellular state of stress tolerance. Many of these factors are co-expressed in response to a stimulus and therefore genomic scale investigations using either microarray or cDNA sequencing are often helpful in their identification. One of the recent approaches used for whole-genome identification of transcripts is RNA-Seq, which relies on sequencing small stretches of RNA-derived cDNAs at a very high coverage. The small sequences are later assembled with advanced computing tools to reconstruct the transcript. As RNA-Seq provides an absolute measure of the quantity, it can be used to deduce the relative expression of a transcript in two different tissues/conditions. Additionally, because RNA-Seq is an open-ended approach, it has been widely used to sequence and assemble *de-novo* transcriptome of various organisms [7-9].

*Brassica juncea* (Czern) L. (AABB, 2n = 36) commonly known as ‘Indian mustard’ is an important oilseed crop. It is a natural amphidiploid species that originated from a cross between *B. rapa* (AA, 2n = 20) and *B. nigra* (BB, 2n = 16). It is widely grown in India, Canada, Australia, China and Russia [10-13]. Considering its economic importance, efforts has been undertaken to augment its economically and agronomically significant traits like oil content, oil quality, seed size, pod shattering and pathogen resistance [14-21]. However, only a few studies have addressed the effects of abiotic stresses in *Brassicas* [22,23]. In Indian subcontinent an early sowing and harvesting of Indian mustard is preferred so that the crop can be harvested before the onset of detrimental aphid attack. Due to an increase in mean temperatures globally, many a times in India, farmers shift sowing of *B. juncea* from October to November and render the crop to aphid attack during it’s maturation. Cultivars of *B. juncea* whose seedlings can germinate efficiently under higher temperatures (which are sometimes encountered during the month of October) can help in escaping the aphid attack as these cultivars can be harvested before the onset of such an attack. The water footprint of *B. juncea* is very small as compared to most of the other cash crops of India, nevertheless, seedling emergence and its sustainability are severely hampered under severe drought conditions [24,25]. Additionally, incidences of high temperature and drought stress during pod development are known to reduce seed setting [26,27]. To fully comprehend the response of *B. juncea* we sequenced and assembled transcriptome of its seedlings that were subjected either to high temperature or drought conditions.

Till now three independent research studies have been carried out to explore the transcriptome of *B. juncea*. Sun et al. [28] performed high throughput sequencing to identify the genes involved in stem swelling in *B. juncea* var. tumida Tsen et Lee, commonly known as tumorous stem mustard [28]. Sequencing of RNA-Seq libraries obtained from different developmental stages of stem of two contrasting strains namely, Yong’an (having inflated tumorous stems) and Dayejie (without inflated stems) generated approximately 54 million reads. Nearly 0.14 million unigenes were predicted out of which around one thousand genes were differentially expressed in the six comparison groups. In another study, Liu et al. [29] investigated seed coat related transcriptome in *B. juncea* varieties Sichuan Yellow Seed (SY) and its brown-seeded near-isogenic line A (NILA) [29]. They identified 69605 unigenes out of which 46 were shown to be involved in flavonoid biosynthesis pathways. Recently, Paritosh et al. [30] explored transcriptome of *B. juncea* var. Varuna (representing the Indian gene pool) and *B. juncea* var. Heera (representing the east European gene pool) to catalogue existing single nucleotide polymorphisms (SNPs) in the two distantly related varieties. Nearly 0.13 million SNPs were identified among which 85473 belong to “A” genome and 50236 are present in “B” genome. These SNPs can be utilized for fine mapping of agronomically important traits and will shed light on the diversification of *Brassica* species [30]. As per our understanding abiotic stress related transcriptome investigations have not been carried out in *B. juncea*. However, such studies have been performed in closely related *B. rapa* and *B. napus* [22,23]. Yu et al. [23] performed RNA-Seq of drought stressed *B. rapa* plants to analyze changes in its transcriptome. Analysis of sequenced tags identified 1092 dehydration responsive genes, many of which were transcription factors [23]. In another study by Zou et al. [22], genome-wide gene expression changes were identified under waterlogging stress in ZS9, a waterlogging-tolerant variety of *B. napus*. High-throughput sequencing of the libraries generated approximately 30 million reads. Data analysis of these libraries revealed presence of 4432 differently expressed genes between the control and waterlogged sample [22].

In the present study we performed high throughput sequencing of the coding transcriptome in *B. juncea* seedlings that were challenged either with high temperature or drought stress. More than 180 million purity filtered reads were used for *de-novo* assembly resulting in identification of approximately 97000 unique transcripts. Nearly 69,245 transcripts were annotated out of which 2834 were kinases and 886 were transcription factors (TF). Expression analysis revealed that 19110 transcripts were differentially regulated by either high temperature and/or drought stress as compared to the control sample. Amongst the differentially expressed transcripts were 92 TFs whose expression changed in response to high temperature. Similarly, drought stress resulted in a significant change in steady state levels of 72 TFs. Moreover, 60 TFs were regulated by both high temperature and drought stress. Among the up-regulated TFs, HSF and DREB constituted the most responsive TF families in BHS and BDS, respectively. Significant alterations in levels of 669 protein kinases by elevated temperature and water deprivation were also noticed. We observed that 259 and 217 protein kinase genes were specifically regulated by drought and high temperature, respectively. A substantial number of kinases (193) were regulated by both high temperature and drought. Role of differentially regulated transcripts was analyzed by their corresponding gene ontologies. Furthermore, we were able to map 1854 of the differentially regulated transcripts in 239 metabolic pathways. Our study not only provides a transcriptome resource that can be utilized for improvement of *B. juncea* and related crops but also improves realm of our existing knowledge for high temperature and drought regulated genes at a genome-wide level.

## Results

### High throughput sequencing, quality filtering and de-novo assembly

Three transcriptome libraries were constructed using Poly A^+^ RNA isolated from hydroponically grown 7-day old whole seedlings that were kept under controlled conditions (BC) or challenged with high temperature (BHS) or drought (BDS). High throughput sequencing of transcriptome libraries using Illumina GA IIx platform generated an aggregate of 183.7 million purity filtered reads amounting to 15.2 Gb of data. Individually, maximum number of reads was obtained in control (BC; ~77.9 million) followed by high temperature stress (BHS; ~65.6 million) and drought stress (BDS; ~40.1 million) samples. The reads which had adapter contamination and low base quality (≤ Q20) were removed to retain approximately 66.1 million, 51 million and 35.5 million high quality (HQ) reads in BC, BHS and BDS samples, respectively. The number of reads that were eliminated from data so as to retain only the HQ reads is presented in Table 1. Subsequently, the base composition of HQ reads was examined to rule out sequencing bias (Additional file 1: Figure S1).

**Table 1:** **Filtering of raw reads obtained through high throughput sequencing of RNA-Seq libraries**

**Category**	**BC**	**BHS**	**BDS**
	**Number of reads**	**Number of reads**	**Number of reads**
	**(Percentage)**	**(Percentage)**	**(Percentage)**
**Raw reads**	77926818 (100%)	65644688 (100%)	40181314 (100%)
**Adapter contaminated**	155835 (0.2%)	4872907 (7.4%)	889239 (2.2%)
**Low quality**	11662189 (15.0%)	9706889 (14.8%)	3747523 (9.3%)
**High quality paired reads**	58438630 (75.0%)	41320578 (62.9%)	32342960 (80.5%)
**High quality unpaired reads**	7670164 (9.8%)	9744314 (14.8%)	3201592 (8.0%)
**Total high quality reads**	66108794 (84.8%)	51064892 (77.8%)	35544552 (88.5%)

Raw reads from control (BC), high temperature (BHS) and drought (BDS) stress libraries were subjected to various quality control parameters and reads that had contamination of adapter sequence or of low quality were eliminated. Only high quality paired and orphan reads were pooled for assembly.

**Figure 1:**
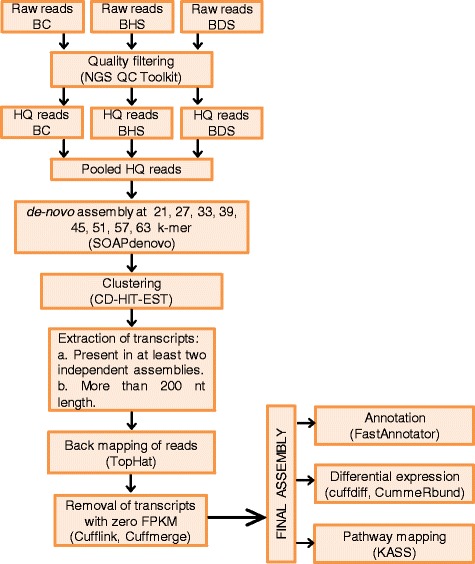
**Schematic overview of the methodology employed for data quality control (QC),** ***de-novo*** **assembly and downstream analysis.** Name of tool used in each step of assembly or analysis is indicated in parenthesis.

**Figure 2:**
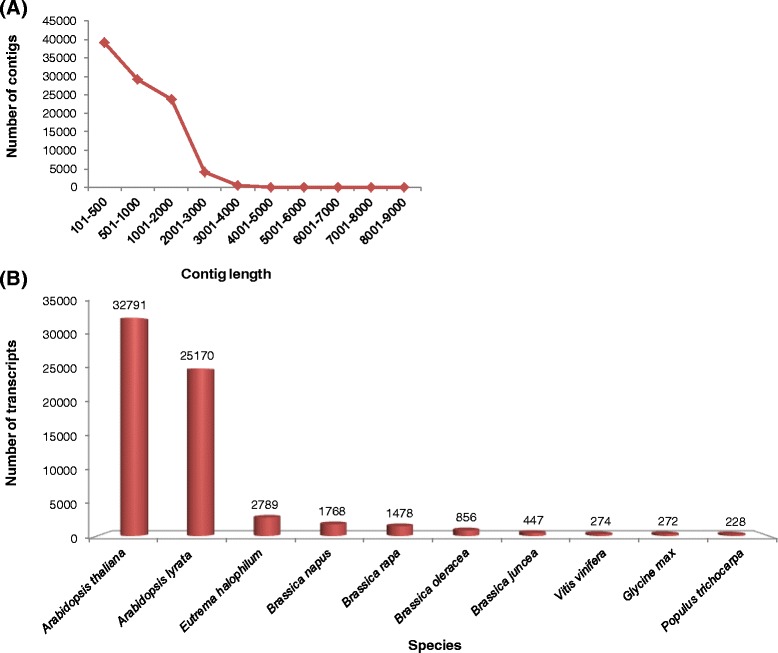
**Investigation of assembly performance and annotation. (A)** Length-wise distribution of contigs. The number of contigs present in each of the length category in clustered transcriptome of *B. juncea* is shown. Contig numbers gradually decreases with respect to increasing contig length. **(B)** Number of *B. juncea* transcripts (Y-axis) that were annotated on the basis of homology with genes from closely related species (X-axis). Transcripts were searched against EMBL plant protein database and based on BLAST score annotations were derived for each transcript. The number of transcripts hitting the protein dataset of various plant species is indicated.

To generate a comprehensive assembly, HQ reads from all the libraries were pooled generating a population of nearly 152.7 million reads. Due to unavailability of assembled genomic sequence in *B. juncea*, reads were ‘*de-novo*’ assembled using SOAPdenovo [31]. The overall strategy of *de-novo* assembly by utilizing HQ reads is presented in Figure 1. Data was independently assembled with different K-mer lengths of 21, 27, 33, 39, 45, 51, 57 and 63 bases. The consolidated results of the assembled data obtained for each of the above K-mers are presented in Table 2. Maximum numbers of contigs (262233) were obtained at 33 K-mer, whereas assembly at 39 K-mer yielded the highest output of 111.6 million bp. As expected, length of the longest assembled transcript gradually decreased with an increasing K-mer for e.g. length of longest transcript was 12248 bp at 27 K-mer and was 7678 bp at 63 K-mer. Average transcript length of 724 bp at 57 K-mer was the best amongst all assemblies. We also evaluated the N50 value and assemblies performed at longer K-mers (39 mer onwards) had a better N50 value than the lower K-mer assemblies. Highest N50 value of 1301 bp was obtained in 51 K-mer assembly. An aggregate of approximately 0.8 million contigs were obtained from all the assemblies. However, significant number of the contigs were represented in only one of the K-mer assemblies and were discarded thereby reducing the number from 0.8 million to 0.27 million. To further filter out the low confidence transcripts, we discarded the contigs that had less than one fragment per kilobase per million (FPKM) in all the conditions (BC, BHS and BDS). In this way, we clustered only those contigs which were present in assemblies of at least two different K-mer and on which at least one fragment out of one million sequenced reads mapped per kilo base. Applying these criteria 97175 contigs with an average length of 817 bp were identified (Table 3). The aggregate length of all the assembled contigs was 79407853 bases. A large percentage (40.3%) of the contigs was in the size range of 100–500 bp. As shown in Figure 2A, the number of contigs decreased with an increasing size range (Figure 2A and Additional file 2: Table S1).

**Table 2:** **Assembly statistics of high quality reads**

**Parameters**	**K-mer**							
	**21**	**27**	**33**	**39**	**45**	**51**	**57**	**63**
**Number of contigs**	204991	248954	***262233***	220102	170941	134378	99899	68700
**Assembly length (million bp)**	69.8	96.1	111.1	***111.6***	104.4	91.9	72.4	47.0
**Minimum transcript length (bp)**	100	100	100	100	100	100	100	100
**Maximum transcript length (bp)**	10071	***12248***	11901	11782	11856	9105	8870	7678
**Average transcript length (bp)**	340	385	423	506	610	683	***724***	684
**N50 (bp)**	665	832	989	1144	1265	***1301***	1241	1057

Pooled high quality reads were assembled at various K-mers using SOAPdenovo. For each of the K-mer various assembly parameters (such as number of contigs, assembly length, minimum, maximum and average transcript length and N50) were evaluated. The maximum value for each of the parameter in their respective k-mers has been italicized.

**Table 3:** **Output of clustered assembly**

**Category**	**Clustered assembly**
**Number of contigs**	97175
**Assembly length (million bp)**	79.4
**Average transcript length (bp)**	817

Assemblies from all the K-mer lengths were subjected to clustering. The number of contigs after clustering, total length of assembly and average length of transcripts is shown.

### Functional annotation of assembled transcripts

*De-novo* assembly followed by clustering resulted in approximately 97000 contigs. Any contig less than 200 bp long was removed from the clustered data thereby reducing the number of contigs to 77750, which were subsequently used for homology-based annotation. Annotation on one hand helps in predicting the functions and on the other hand provides confidence about assembly approach. A substantial portion of the assembled contigs would be annotated as long as assembly approach is robust and adequate protein information of closely related species is available. These contigs hereafter referred as transcripts were searched against non-redundant protein database of EMBL (European Molecular Biology Laboratory) by using FASTAnnotater tool (http://fastannotator.cgu.edu.tw/) with an e-value cut-off of 0.00001. Also, a query coverage threshold of 70% identity was used to discard low coverage/ambiguous homologous protein mapping. Each transcript was annotated as per the best homologous protein and the corresponding annotation was assigned to it. Based on the above approach 89% (69245) of the transcripts were annotated whereas 11% (8506) transcripts remained unannotated (Additional file 3: Table S2). A total of 25438 transcripts had one or more protein domains based on information of pfam database (http://pfam.xfam.org/). We were able to identify 3895 unique pfam domains (Additional file 3: Table S2). BLAST (Basic Local Alignment Search Tool) score revealed that highest number of transcripts matched to *A. thaliana* (32791) and *A. lyrata* (25170). The number of transcripts that matched with *B. rapa* or other *Brassica* species were less than that of *A. thaliana* and *A. lyrata* (Figure 2B and Additional file 4: Table S3). This observation is in accordance with the fact that protein resource of *Arabidopsis* is much more comprehensive as compared to that of Brassica species.

### Transcriptome analysis in response to high temperature and drought stress: Quantification, differential expression and pathway mapping

**Figure 3:**
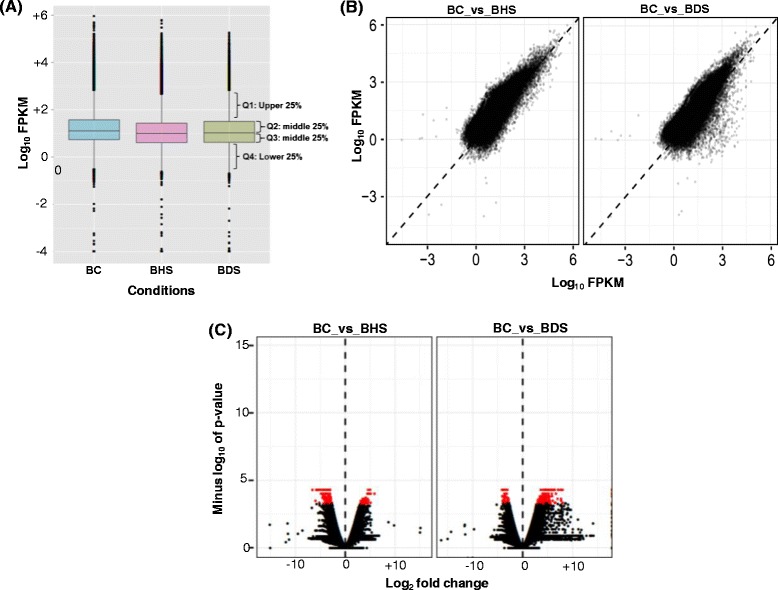
**Estimation of normalization and expression changes in different libraries. (A)** Box-and-whisker plot of log_10_ FPKM values in RNA-Seq libraries of control (BC), high temperature (BHS) and drought stress (BDS). The entire range is divided in 4 quartiles (Q1-Q4) each representing 25% of genes in the particular range. **(B)** Scatter plot and **(C)** Volcano plot of the transcriptome in high temperature (BHS) and drought (BDS) stress. In scatter plot, log_10_ FPKM values in control (X-axis) have been plotted against log_10_ FPKM values of stress treated sample (Y-axis) sample. In volcano plot, statistical significance (−log_10_ of *p*-value; Y- axis) has been plotted against log_2_ fold change (X-axis).

We used FPKM (Fragments Per Kilobase per Million) method to normalize the expression of identified transcripts across different conditions. To visualize the range of transcript abundance, log_10_ values of FPKM were used to construct box-and-whisker plot for each of the condition. As seen in the Figure 3A, majority of the transcripts fall in the log_10_ FPKM range of 0–2. However, many of the transcripts have log_10_ FPKM values higher and lower than this range. These transcripts are the outliers and are represented by black dots (each dot representing one transcript). It was observed that median and quartile values across BC, BHS and BDS were almost similar. Scatter plots drawn with the log_10_ FPKM values further corroborated the results obtained from box-plots. As seen in Figure 3B, the FPKM values (or in other words the transcript abundance) in both control and stress samples are similar for most of the transcripts. To see how many transcripts are significantly regulated, volcano plots were constructed by plotting the fold change values against the negative log of *p*-values (Figure 3C). The higher the negative log *p*-values, more is the significance of the regulation. In the center of the volcano is a line at which fold change is zero. On one side of the line are the negative fold change values indicating down-regulation and on the other side are the positive fold change values thereby indicating up-regulation. Significantly regulated genes are represented by red dots. As has been shown by many previous studies, our data also follows the similar pattern that a small proportion of all genes are significantly regulated by abiotic stresses [22,23].

**Figure 4:**
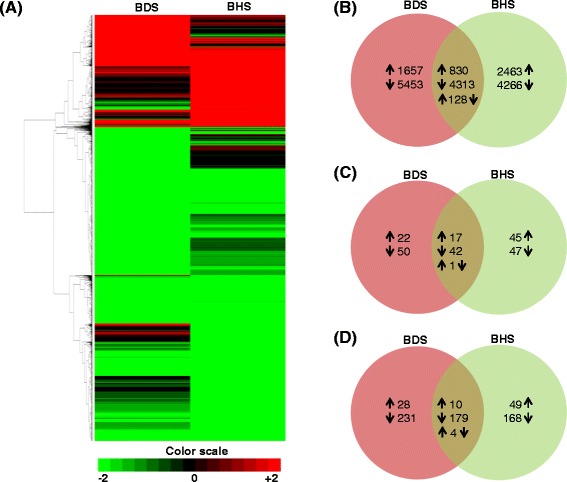
**Expression analysis of differentially expressed transcripts. (A)** Unsupervised hierarchical clustering of differentially expressed transcripts in high temperature (BHS) and drought stress (BDS) conditions. Comparison was made against control sample using Pearson uncentered algorithm with an average linkage rule to identify clusters of genes based on their expression levels across samples. **(B)** Number of transcripts **(C)** transcription factors and **(D)** kinases that were regulated by high temperature stress, drought stress or by both stresses. The up-regulation, down-regulation and inverse corelation (up-regulated in one condition and down-regulated in other condition or *vice versa*) is indicated by arrows pointing upwards, downwards and upwards-downwards, respectively.

To find out the differentially expressed genes FPKM values were compared in stress versus control conditions. A criterion of ± two fold change (on log_2_ scale) was applied and 19110 transcripts were identified that were regulated at least 2 folds in either high temperature stress and/or drought stress. Out of 19110 transcripts, 5271 were regulated by both stresses whereas 6729 and 7110 were regulated specifically by high temperature (BHS) and drought (BDS) stress, respectively. Upon imposition of stresses, majority of transcripts were down-regulated. Out of 19110 significantly regulated transcripts, 14032 were down regulated, 4266 of which were specifically down-regulated by high temperature stress, 5453 by drought stress and 4313 by both high temperature and drought stress. A heat map of differentially regulated transcripts is presented in Figure 4A. The heat map clearly shows that a greater number of transcripts are down regulated as compared to up regulated transcripts. Nevertheless, a lesser but substantial number of the transcripts were up regulated too, for example in BHS 2463, in BDS 1657 and in both BHS and BDS 830 transcripts were up regulated (Figure 4B). Interestingly, 128 transcripts regulated by both BHS and BDS displayed an inverse correlation in their expression with respect to these two stresses. Details of differentially regulated transcripts are provided in Additional file 5: Table S4.

We also looked into the pathways in which the differentially expressed genes are involved. We were able to map 1854 genes in 239 different metabolic pathways (Additional file 6: Table S5). To further narrow down on the most significant pathways, we shortlisted the pathways in which at least 10 differentially regulated genes were present. Based on the above criteria 51 significant pathways were shortlisted. The maximum numbers of differentially regulated genes (87) were present in ‘ABC transporters’, followed by ‘ribosome biogenesis’ having 76 genes and ‘purine metabolism’ with 43 genes. A list of top 10 metabolic pathways possibly regulated by high temperature and/or drought stress is presented in Table 4. For each of the pathway, the hierarchical categorization of KEGG (Kyoto Encyclopedia of Genes and Genomes) identifier in the form of KEGG BRITE has also been included in the table.

**Table 4:** **List of top 10 dysregulated pathways**

**KEGG ID**	**Pathway**	**BRITE Class-1**	**BRITE Class-2**	**Number of transcripts**
**ko02010**	ABC transporters	Environmental Information Processing	Membrane transport	87
**ko03010**	Ribosome	Genetic Information Processing	Translation	76
**ko00230**	Purine metabolism	Metabolism	Nucleotide metabolism	43
**ko00860**	Porphyrin and chlorophyll metabolism	Metabolism	Metabolism of cofactors and vitamins	41
**ko00010**	Glycolysis/Gluconeogenesis	Metabolism	Carbohydrate metabolism	37
**ko00520**	Amino sugar and nucleotide sugar metabolism	Metabolism	Carbohydrate metabolism	36
**ko02020**	Two-component system	Environmental Information Processing	Signal transduction	36
**ko00520**	Amino sugar and nucleotide sugar metabolism	Metabolism	Carbohydrate metabolism	34
**ko00540**	Lipopolysaccharide biosynthesis	Metabolism	Glycan biosynthesis and metabolism	33
**ko00230**	Purine metabolism	Metabolism	Nucleotide metabolism	31

Differentially regulated transcripts were mapped on various metabolic pathways using corresponding KEGG identifiers. Derived pathway and associated BRITE Class with number of dysregulated genes are indicated.

### Gene ontology analysis of stress-regulated transcripts

**Figure 5:**
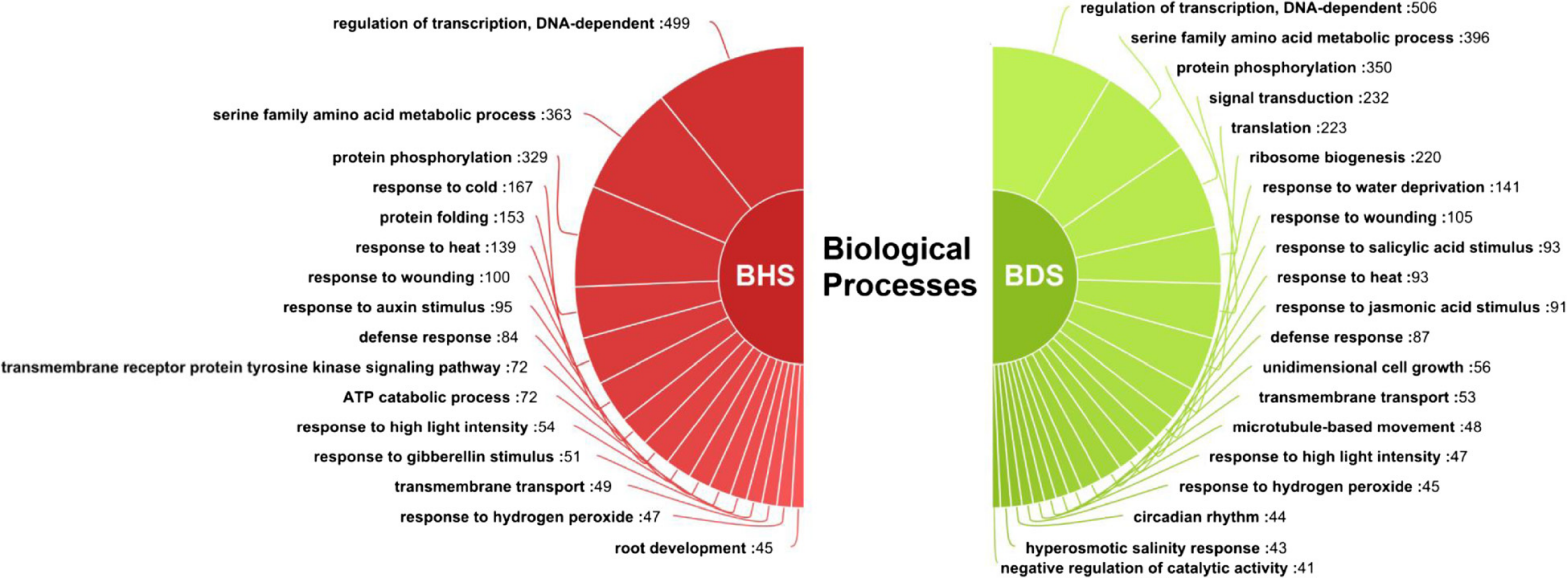
**Gene ontology classification of differentially expressed transcripts under the ‘biological process’ category.** Significant GO terms (having atleast 40 genes) associated with differentially expressed transcripts in high temperature (BHS) and drought (BDS) stress samples along with the number of genes is indicated.

For a broader classification, the entire set of 19110 stress-modulated transcripts was subjected to gene ontology (GO) analysis. Nearly 40% of high temperature stress and 43% of drought stress regulated genes were associated with the GO category ‘biological process’. Similarly, 34% and 31% of the high temperature and drought stress regulated genes were linked with ‘molecular function’ category, respectively. Further, 26% of genes regulated by either high temperature or drought stress were placed in ‘cellular component’ category. A significant number of transcripts (499 in BHS and 506 in BDS) were categorized under the GO number ‘GO:0006355’ representing ‘regulation of transcription’. Other apparent GO terms associated with differentially expressed genes were ‘serine family amino acid metabolic process (GO:0009069)’ and ‘protein phosphorylation (GO:0006468)’. More than 300 transcripts associated with each of the above-mentioned GO category. For each of the stress conditions, a few GO terms, for example, ‘response to heat (GO:0009408)’ and ‘response to high light intensity (GO:0009644)’ were enriched in high temperature stress library. In case of drought stress treated library, the enriched GO terms included ‘response to water deprivation (GO:0009414)’ and ‘hyperosmotic salinity response (GO:0042538)’. The composition of significant GOs, having more than 40 differentially regulated genes, in BDS and BHS samples is presented in Figure 5.

Hormones play an important role in defining plant’s response to high temperature and drought stress [32-34] and therefore, many GO terms related to hormone signaling were enriched from the genes regulated by heat and/or drought stress. Some of the enriched categories were ‘response to auxin stimulus (GO:0009733)’, ‘response to salicylic acid stimulus (GO:0009751)’, response to ‘jasmonic acid stimulus (GO:0009753)’, ‘abscisic acid transport (GO:0080168)’ and ‘response to gibberellin stimulus (GO:0009739)’. Approximately, 2914 and 2458 stress modulated transcripts from BDS and BHS samples respectively, were associated with the top 20 GO terms (Additional file 7: Table S6, Additional file 8: Table S7).

### Expression analysis of transcription factors and protein kinases

Considering the functional importance of transcription factors and protein kinases, we identified 886 transcription factors and 2834 protein kinases in the assembled *B. juncea* transcriptome (Additional file 9: Table S8, Additional file 10: Table S9). A large collection of transcription factor families and their members have been reported in Arabidopsis [35]. Similarly, we also discovered multiple members of transcription factor families in our data, including 122 transcripts belonging to MYB family. Other abundant transcription factor family members were from WRKY (118), bHLH (101), CCAAT (48), HSF (39), NFY (37), JUMONJI (37), AP2 (32), GATA (29), ERF (26), C2H2 (22), PLATZ (21), bZIP (21), DREB (15). Amongst the protein kinases, maximum numbers of transcripts (240) were identified for receptor-like kinase family. Beside these, MAP kinases (116), casein kinases (80), calcium-dependent protein kinases’ (62), CBL-interacting protein kinases (59) and cyclin-dependent protein kinases (40) were also represented abundantly in the assembled transcriptome data.

Following identification of TFs and kinases, we ascertained their digital expression so that they can be catalogued on the basis of their modulation by stress. Our analysis revealed that expression of 72 and 92 TFs changed by at least log_2_ ± 2 folds in response to drought and high temperature stress, respectively. Additionally, expression of 60 TFs changed significantly by both the stresses (Figure 4C). It was noticed that among the differentially regulated transcription factors in high temperature stressed sample most dominating category was of MYB-transcription factors (26) followed by HSF (23) and ERF (15). Together these three classes of transcription factors represent 25% of all the transcription factors that were differentially regulated by heat stress. In case of transcription factors responsive to drought stress, MYB transcription factors constitutes largest group (17) followed by bHLH (13) and WRKY (12) transcription factor members. When we searched for the TFs, whose expression was significantly up-regulated, we observed that HSF family (21 members) and DREB family (7 members) were the predominant families in high temperature and drought stress, respectively. Similarly, investigation of abundances of protein kinases revealed change in expression of 669 kinases with respect to their expression in control sample. Among the various kinase families, 86 members of receptor-like kinase, 29 members of MAP kinase, 15 members of casein kinase, 11 members of calcium-dependent kinase, 6 members each of CBL-interacting kinase and cyclin dependent kinase families were regulated by more than two fold. Moreover, out of 669 differentially regulated kinases, 259, 217 and 193 were regulated by drought, high temperature or both stresses, respectively (Figure 4D). These results indicate that heat and drought stress drive change in expression of many transcription factors and kinases which serve as key components of signal transduction pathways. Some of these are regulated by both stresses while others are specifically involved in either heat or drought stress response. The number of differentially regulated transcripts of various transcription factor and kinase families is presented in Table 5. Information about the individual transcripts can be found in Additional file 9: Table S8 and Additional file 10: Table S9.

**Table 5:** **Differential expression of transcripts annotated as transcription factors and kinases**

**Family**	**Unique in BHS and/or BDS**		**BDS**			**BHS**		
	**Transcripts identified**	**Differentially expressed transcripts**	**Up regulated**	**Down regulated**	**Total**	**Up regulated**	**Down regulated**	**Total**
Transcription factors								
MYB	122	34	4	13	17	12	14	26
HSF	39	24	7	2	9	21	2	23
ERF	26	22	2	9	11	6	9	15
WRKY	118	21	5	7	12	3	11	14
bHLH	101	18	1	12	13	1	9	10
AP2	32	14	4	2	6	5	7	12
DREB	15	11	9	0	9	10	0	10
JUMONJI	37	8	0	7	7	0	4	4
GATA	29	7	0	5	5	2	2	4
bZIP	21	6	1	4	5	0	3	3
PLATZ	21	4	3	0	3	1	0	1
TCP	8	3	1	0	1	1	0	1
CCAAT	48	2	0	1	1	0	1	1
HD	5	2	0	1	1	0	1	1
SCARECROW	5	1	0	1	1	0	1	1
GRAS	5	1	1	0	1	1	0	1
NFY	37	0	0	0	0	0	0	0
C2H2	22	0	0	0	0	0	0	0
Kinases								
Receptor-like kinases	240	86	4	59	63	2	52	54
MAP kinases	116	29	6	14	20	2	10	12
Casein kinases	80	15	1	9	10	2	7	9
Calcium-dependent protein kinases	62	11	2	7	9	1	8	9
CBL-interacting kinases	59	6	0	3	3	1	3	4
Cyclin-dependent kinases	40	6	0	6	6	0	3	3

The members of various transcription factor and kinase families were fetched from assembled transcriptome data and analyzed for expression pattern under conditions of drought (BHS) and high temperature (BHS). The details of total and differentially regulated transcripts in respective families along with categorization as up-regulated, down-regulated and total regulated transcripts in BDS and BHS is presented.

### Validation of differentially regulated transcripts

**Figure 6:**
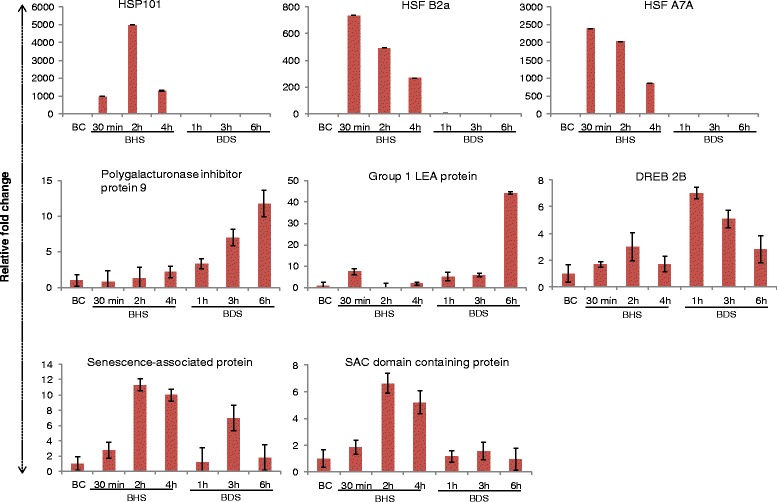
**Relative abundance of selected transcripts as determined by qPCR.** Expression profiling of a few differentially regulated transcripts was performed using quantitative real time PCR. The relative abundance (Y-axis) was calculated using ΔΔCt method. *B. juncea* seedlings were subjected for varied durations to either high temperature stress (BHS) at 42°C for 30 min, 2 h and 4 h or drought stress (BDS) by using 300 mM mannitol for 1 h, 3 h and 6 h. The mean of three independent biological replicates is presented.

From the list of significantly regulated transcripts, eight transcripts were selected for experimental validation and expression profiling. These transcripts include TCONS_00034159, TCONS_00057510, TCONS_00068803, TCONS_00031582, TCONS_00018135, TCONS_00075263, TCONS_00034464 and TCONS_00054852 which were annotated as HSP101, HSFB2a, HSFA7a, DREB2B, group 1 LEA protein, polygalacturonase inhibitor protein 9, SAC-domain containing protein and senescence associated protein, respectively. As expected expression of HSP101, HSFB2a and HSFA7a increased substantially and specifically in high temperature stress treatment whereas genes encoding for DREB 2B, Group 1 LEA protein and polygalacturonase inhibitor protein 9 were induced by drought stress. A significant increase in the expression of Group 1 LEA protein was also observed in high temperature stress. SAC-domain containing protein and senescence-associated protein were inducible by both high temperature and drought treatment. The relative expression profiles of the above mentioned transcripts are depicted in Figure 6.

## Discussion

Ecological confinement of crops is determined by the climatic conditions prevailing in a niche. Ever-increasing population and decreasing arable land is straining economies of the countries that are largely dependent on agronomic produce. Multiple abiotic factors that act either in isolation or combination contribute to decrease in overall yield of crops. Amongst abiotic factors, high temperature and water scarcity has an implacable effect on plant physiology and undermines the plant’s capability to sustain adequate grain production. To mitigate the effects of stress injuries, it is critical to contrive plants that can withstand environmental challenges. Identification of molecular factors that either reinforce or provide *ab initio* abilities to combat these stresses is therefore of paramount importance.

The primary objective of this study was to visualize the landscape of changes occurring in transcriptome of *B. juncea* upon imposition of high temperature and drought stresses. Here, we carried out paired end sequencing of RNA-Seq libraries prepared from poly A^+^ RNA isolated from hydroponically grown 7-day old seedlings that were either grown under control conditions or subjected to high temperature and drought stress. High throughput sequencing generated more than 180 million purity filtered reads and nearly 150 million HQ reads were *de-novo* assembled using SOAPdenovo assembler. Assembly was performed at multiple K-mers and assemblies obtained from all the K-mers were clustered together. We adopted assembly at multiple K-mers primarily because of two reasons: firstly, many studies have shown that *de novo* assemblies with multiple K-mers result in discovery of greater number of transcripts [36,37] and secondly it provides an opportunity to remove the contigs that are present in only one of the K-mer assembly, thereby increasing the confidence on the assembly. Data assembled with multiple K-mers was clustered, followed by removal of singletons. Subsequently, the resultant transcriptome was analyzed by assigning annotations, expression (FPKM values), gene ontologies and other functional categories. Based on the digital expression data many transcripts regulated by either high temperature and/or drought were shortlisted.

We report the existence of more than 97000 unique transcripts in Indian mustard. However, a significant proportion of these unique transcripts were smaller than 200 bases. Suspecting that these are artifacts of *de-novo* assembly, we discarded them to obtain 77750 unique transcripts. The fact that a large number of assembled transcripts were annotated provides another support for the multi K-mer approach adopted for assembly. Analysis of expression patterns of these transcripts revealed, 19110 unique transcripts were responsive to drought stress and/or high-temperature. Moreover, 5271 transcripts were regulated (830 up regulated, 4313 down regulated, 128 with inverse regulation) by both high temperature and drought stress. Several studies have previously shown that some components are involved in more than one stress-signaling pathway [38-45] and therefore functional characterization of the transcripts that are up regulated by both these stresses will shed light on the conserved signaling pathways in *B. juncea*. Equally important are the transcripts that display an inverse correlation with respect to these stresses, as their characterization will help us unravel the reasons for their inverse regulation and functional significance.

Of the genes identified in our study are the TFs like DREBs, HSFs, WRKYs, MYBs etc. and calcium sensors, kinases, calmodulin-binding chaperonins, glutathione transferases, ascorbate peroxidases, ferritin etc. many of which have previously been implicated, in multiple abiotic stresses including drought and high temperature [46-51]. A detailed investigation of the digital expression data revealed that 7110 and 6729 genes were modulated specifically by drought and high temperature stress, respectively. As reported previously in multiple studies a majority of these genes were down regulated upon stress imposition indicating a general transcriptional repression [52]. Of the 19110 stress- modulated transcripts 1854 mapped onto different metabolic pathways, the few significant of which included “ABC transporters”, “purine metabolism”, and “two component systems”. Components of the above-mentioned pathways are involved in abiotic stresses and therefore it is plausible that the *B. juncea* transcripts mapping to these pathways also play an important role in mitigating effects of abiotic stresses. At the center of abiotic stress signaling are TFs and kinases many of which are themselves regulated by abiotic stresses. Our data reveals presence of 886 TFs and 2834 kinases, out of which 256 TFs and 669 kinases were regulated by high temperature and drought stress respectively. The major up-regulated TFs in high temperature and drought stress turned out to be *HSFs* and *DREBs*, which are the known biomarkers for these stresses, respectively.

In order to prove the authenticity of *B. juncea de-novo* assembly, we selected a few transcripts and validated them using quantitative real time PCR. Three of the shortlisted targets were *HSP101*, *HsfB2a* and *HsfA7a*, homologues which show a specific induction by heat stress. Time kinetics studies of *B. juncea HSP101*, *HsfB2a* and *HsfA7a* shows that these transcripts are induced many folds under high temperature [53-57]. Moreover, the induction of the TFs HSFB2a and HSFA7a precedes that of HSP101 indicating a hierarchy in stress signaling. Another transcript, which was validated by QPCR, was a member of group I LEA protein that are known to accumulate in water deprived cells [58,59]. As expected expression of LEA transcript increased nearly 40 folds under sustained conditions of drought. Surprisingly, approximately, 10-fold induction of LEA transcript was observed in high temperature stressed seedlings also. Reports suggest that LEA proteins can act synergistically with trehalose to prevent protein aggregation *in vitro* during high temperature [60]. *In-vivo* trehalose accumulates in plants subjected to high temperature stress [43,61,62] and hence it is conceivable that the accumulated LEA proteins act in conjunction with trehalose to *in-vivo* obviate the protein denaturation occurring during high temperature stress. Polygalacturonase inhibiting proteins (PGIP) are synthesized in plants to inhibit the activity of polygalacturonase enzyme secreted by phytopathogenic fungi [63]. *AtPGIP1* is inducible by cold stress [63] and analysis of 27 different PGIPs revealed that abiotic stress responsive cis-regulatory elements are present in their promoters [64]. Induction of PGIP under drought stress in the present study thereby indicate that PGIP is involved in multiple biological processes and may provide a link between drought stress mediated signaling and plant defense response. SAC domain containing proteins were initially discovered in yeast and are believed to act as phosphoionositide phosphatases. Arabidopsis has 9 SAC domain containing proteins and *AtSAC6* is inducible by salinity stress [65]. We believe that multiple SAC domain containing proteins are present in *B. juncea* and induction of some of the members in abiotic stresses might be helpful in attenuating stress signaling by removing phosphate from phosphoionositides.

## Conclusion

In present study we have utilized next generation sequencing and computational methods to decipher the genome-wide perturbations of steady state levels of transcripts in *B. juncea* seedlings subjected to high temperature and drought stress. We identified more than 97000 transcripts out of which approximately 19000 were differentially regulated. Importantly, we also identified multiple TFs and protein kinases that were modulated by these stresses. These transcripts are components of important physiological processes, signaling/metabolic pathways and regulatory networks. Stress responsive genes identified in this study will be useful in expanding our knowledge of high temperature and drought stress biology. The identified transcripts can be used to engineer tolerance against two of the most important abiotic stresses in *B. juncea* and related crop species.

## Methods

### Plant material and growth conditions

Seeds of *Brassica juncea* var. Varuna were obtained from National Seed Center (NSC), Indian Agricultural Research Institute (IARI), Delhi, India. Seeds were surface sterilized with 2% sodium hypochlorite solution for 10 minutes (min) on a shaker and then washed five times with double distilled water for three min each. Sterile seeds were hydroponically grown on a muslin cloth wrapped around a small container in a growth chamber at 24°C ± 1 with 16 hours (h) day/8 h night photoperiod.

### Stress conditions and treatments

Seedlings were grown for seven days and then subjected to various abiotic stresses. Drought stress was imposed for 3 h and 12 h by replacing water with high osmolality solution (300 mM mannitol). For imposing high temperature stress, seedlings were placed in a BOD incubator (Scientific systems, India) at 42°C for 30 min and 4 h. Entire seedlings (including the roots) were harvested after specified time intervals, snap frozen in liquid nitrogen and stored at −80°C. Untreated seedlings were taken as control.

### RNA isolation, RNA-Seq library preparation and sequencing:

Total RNA was isolated using GITC-based method [66] from abiotic stress treated and untreated whole seedlings, independently for each time point. Extracted RNA was quantified using spectrophotometer (Biorad, USA) and an aliquot of heat denatured RNA was electrophoresed on denaturing agarose gel to check its integrity. RNA extracted from two different time points were pooled in equimolar amounts and three RNA-Seq libraries- BC (control seedlings), BDS (drought stressed seedlings) and BHS (high temperature stressed) were prepared utilizing NEBNext RNA-Seq library preparation Master Mix Set for Illumina procured from NEB, USA. Briefly, Poly A^+^ RNA was isolated from 10 μg of total RNA using Sera-Mag beads (GE Healthcare, UK) and fragmented chemically at high temperature. Fragmented RNA was qualitatively and quantitatively checked on Bioanalyzer (Agilent, USA). 250 ng of fragmented RNA was used for first strand reverse transcription using random primers followed by second strand synthesis. The ends of double stranded cDNA were repaired and mono-adenylated. Paired end adapters were ligated using Rapid T_4_ DNA ligase and then size fractionated. Approximately, 350 bp size region was eluted and PCR amplified for 12 cycles. The quality and quantity of prepared libraries was evaluated utilizing Bioanalyzer (Agilent, USA). Ultra-deep parallel sequencing was performed using Illumina Genome Analyzer IIx at University of Delhi South Campus, Delhi, India according to manufacturer’s instructions.

### RNA-Seq data processing, *de-novo* assembly and annotation

RNA-Seq raw reads were processed by NGS-QC toolkit [67] and low-quality as well as adapter-contaminated sequences were discarded. High quality (paired and unpaired) reads were assembled *de-novo* using SOAPdenovo assembler [31] independently at eight different K-mers (21, 27, 33, 39, 45, 51, 57, 63). The eight assemblies were subsequently clustered by using CD-HIT-EST [68]. The clustering parameters used were ≥80% query coverage and ≥80% identity. To further clean the data transcripts present in only one of the K-mer assemblies were removed. This was followed by removal of transcripts with less than 1 FPKM in all the three conditions (BC, BDS and BHS). Finally all the transcripts less than 200 bp were removed and the remaining transcripts were functionally annotated using FASTAnnotater tool (http://fastannotator.cgu.edu.tw/) with an e-value cut-off of 0.00001 by taking non-redundant protein database of EMBL (European Molecular Biology Laboratory) as a reference. Gene ontology analysis of transcripts was derived through Uniprot hit accessions and prediction of biochemical pathways was performed by KEGG identifiers (http://www.genome.jp/kegg/).

### Quantitative real time PCR validation of differentially expressed genes (DEGs)

Ten microgram of total RNA was treated with two units of RNase free DNase I (NEB, USA) followed by phenol chloroform extraction and precipitation. Two μg of DNase free RNA was reverse transcribed using iScript reverse transcription kit (Biorad Inc., USA). The first strand cDNA was diluted 10 times and used as template. Quantitative real time PCR was performed on CFX connect real time system (Biorad Inc., USA) using gene-specific forward and reverse primers (Additional file 11: Table S10) and SYBR green chemistry (Roche, GmbH). Actin was used as an internal reference gene. Delta delta ct method was used to calculate relative fold change values. Three biological replicates and two technical replicates were included for each experiment.

### Availability of supporting data

The data discussed in this publication have been deposited in NCBI's Gene Expression Omnibus and are accessible through GEO Series accession number GSE64242 (http://www.ncbi.nlm.nih.gov/geo/query/acc.cgi?acc=GSE64242).

## Additional files

Additional file 1: Figure S1. Frequency (in %) of the individual nucleotides in high quality reads of control (BC), high temperature (BHS) and drought (BDS) RNA-Seq libraries. Additional file 2: Table S1. Distribution of number of clusters in various cluster size ranges. Additional file 3: Table S2. List of identified transcripts with their respective IDs, length, relative fold change, best BLASTx hit to protein database and gene ontologies. Additional file 4: Table S3. Homologue species distribution based on BLASTx results. Additional file 5: Table S4. List of differentially regulated transcripts with their respective IDs, length, relative fold change, best BLASTx hit to protein database and gene ontologies. Additional file 6: Table S5. List of dysregulated metabolic pathways. Additional file 7: Table S6 Gene ontologies associated with drought responsive unique transcripts. Additional file 8: Table S7. Gene ontologies associated with high temperature responsive unique transcripts. Additional file 9: Table S8. List of identified transcription factors with their respective IDs, length, relative fold change, best BLASTx hit to protein database and gene ontologies. Additional file 10: Table S9. List of identified kinases with their respective IDs, length, relative fold change, best BLASTx hit to protein database and gene ontologies. Additional file 11: Table S10. Details of primers utilized for quantitative real time PCR.
