# Supplementary material for: Global insights into high temperature and drought stress regulated genes by RNA-Seq in economically important oilseed crop Brassica juncea
Source: BMC Plant Biol. 2015 Jan 21;15:9. doi: 10.1186/s12870-014-0405-1 (PMC4310166; doi:10.1186/s12870-014-0405-1)
Supplement: Additional file 1: Figure S1. — Frequency (in %) of the individual nucleotides in high quality reads of control (BC), high temperature (BHS) and drought (BDS) RNA-Seq libraries. [file 12870_2014_405_MOESM1_ESM.pptx]

## Slide 1
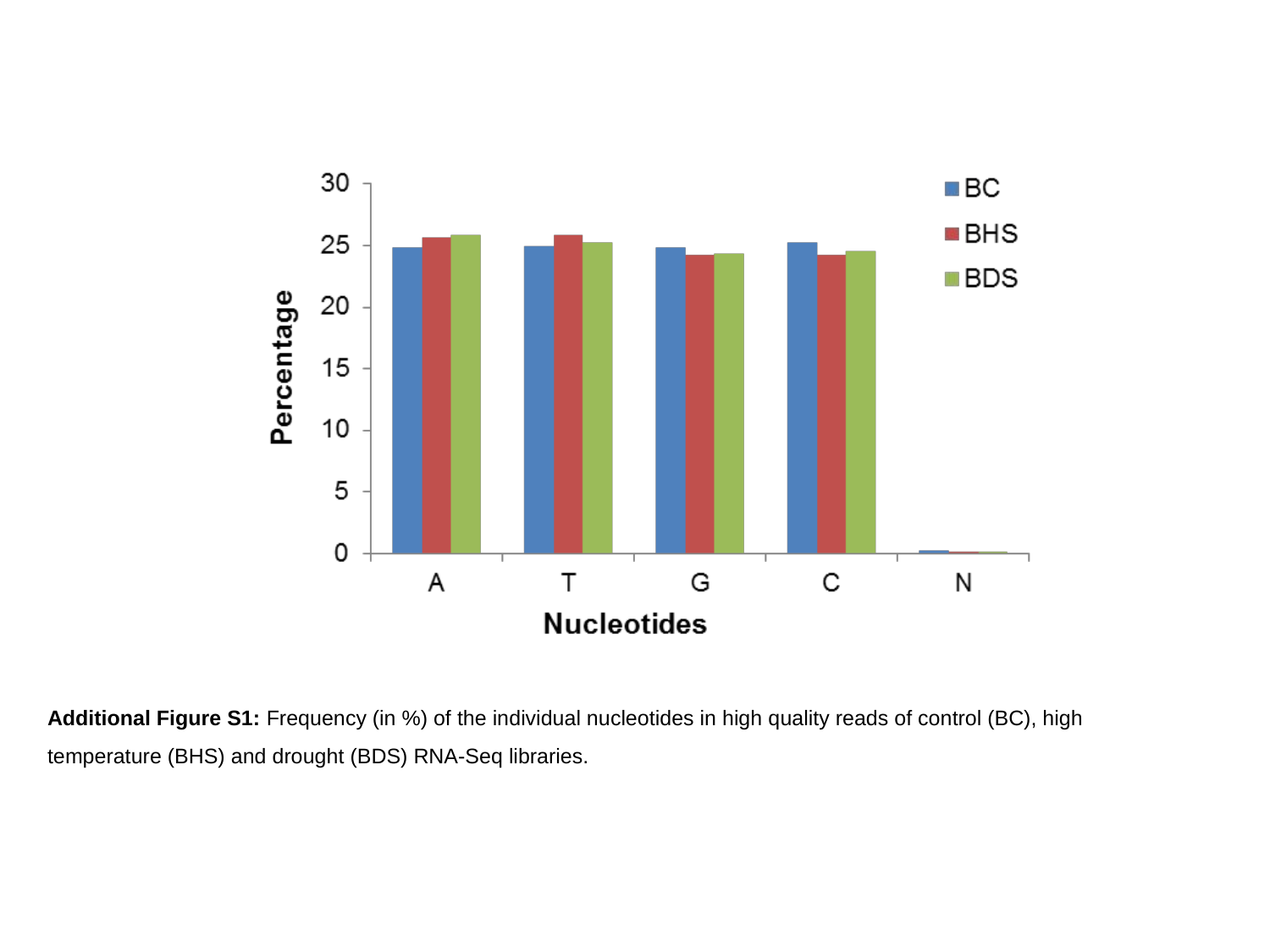

Additional Figure S1: Frequency (in %) of the individual nucleotides in high quality reads of control (BC), high temperature (BHS) and drought (BDS) RNA-Seq libraries.
